# Natural Hazards in a Changing World: A Case for Ecosystem-Based Management

**DOI:** 10.1371/journal.pone.0095942

**Published:** 2014-05-07

**Authors:** Jeanne L. Nel, David C. Le Maitre, Deon C. Nel, Belinda Reyers, Sally Archibald, Brian W. van Wilgen, Greg G. Forsyth, Andre K. Theron, Patrick J. O’Farrell, Jean-Marc Mwenge Kahinda, Francois A. Engelbrecht, Evison Kapangaziwiri, Lara van Niekerk, Laurie Barwell

**Affiliations:** 1 Natural Resources and the Environment, Council for Scientific and Industrial Research (CSIR), Stellenbosch, Western Cape, South Africa; 2 World Wide Fund for Nature (WWF), Cape Town, Western Cape, South Africa; 3 School of Animal, Plant and Environmental Sciences, University of the Witwatersrand, Johannesburg, Gauteng, South Africa; 4 Natural Resources and the Environment, Council for Scientific and Industrial Research (CSIR), Pretoria, Gauteng, South Africa; 5 Centre for Invasion Biology, Department of Botany and Zoology, Stellenbosch University, Stellenbosch, Western Cape, South Africa; Centro de Investigacion Cientifica y Educacion Superior de Ensenada, Mexico

## Abstract

Communities worldwide are increasingly affected by natural hazards such as floods, droughts, wildfires and storm-waves. However, the causes of these increases remain underexplored, often attributed to climate changes or changes in the patterns of human exposure. This paper aims to quantify the effect of climate change, as well as land cover change, on a suite of natural hazards. Changes to four natural hazards (floods, droughts, wildfires and storm-waves) were investigated through scenario-based models using land cover and climate change drivers as inputs. Findings showed that human-induced land cover changes are likely to increase natural hazards, in some cases quite substantially. Of the drivers explored, the uncontrolled spread of invasive alien trees was estimated to halve the monthly flows experienced during extremely dry periods, and also to double fire intensities. Changes to plantation forestry management shifted the 1∶100 year flood event to a 1∶80 year return period in the most extreme scenario. Severe 1∶100 year storm-waves were estimated to occur on an annual basis with only modest human-induced coastal hardening, predominantly from removal of coastal foredunes and infrastructure development. This study suggests that through appropriate land use management (e.g. clearing invasive alien trees, re-vegetating clear-felled forests, and restoring coastal foredunes), it would be possible to reduce the impacts of natural hazards to a large degree. It also highlights the value of intact and well-managed landscapes and their role in reducing the probabilities and impacts of extreme climate events.

## Introduction

Since the turn of the century, several major natural disasters have attracted international attention, including hurricanes Katrina and Sandy, floods in Thailand and the Indian Ocean tsunami. These disasters, along with countless more frequent disasters of smaller magnitude, have been responsible for the loss of at least a million lives over the last decade, with recovery often taking years and financial losses estimated to be in the trillions of US dollars [1]. Such disasters occur when extreme physical events – or ‘natural hazards’ – impact adversely on vulnerable and exposed communities and infrastructure, which are the human elements of disaster [2]. As a result, disaster risk is affected by changes in the incidence of natural hazard, as well as alterations in the patterns of societal exposure and vulnerability. This paper deals specifically with changes in the incidence of natural hazards, which are expected to increase into the future. Changes in climate are expected to result in higher sea levels and increased hurricane activity, bringing more frequent and severe storm-waves that flood and erode coastal areas [2], [3]. In many regions of the world, climate-induced shifts in the water cycle will result in more frequent and intense periods of flooding and drought [4], [5]. Wildfires are also expected to become more widespread and frequent, being closely linked to hot, dry weather conditions and drought [6].

The anticipated increased incidence of natural hazards is not only attributed to climate change. There is a growing concern that rapid and widespread land cover change is leading to the loss of the buffering capacity that healthy ecosystems provide against these natural hazards [7]. For example, healthy mangrove ecosystems, coral reefs and coastal foredunes are able to dissipate wave energy, reducing the impacts of coastal flooding and erosion during storms [8], [9]. Areas with intact mangroves were much less affected by the 1999 and 2004 Indian Ocean tsunamis, than areas where mangroves had been removed [10]. Likewise, healthy inland wetland ecosystems and riparian zones help to absorb peak flows and sediment during extreme rainfall events, reducing flooding and sedimentation hazards to downstream areas [11], [12]. Invasion of natural vegetation by alien trees that use more water than the indigenous vegetation that they replace, exacerbates the effects of water scarcity in drought-prone regions [13], [14]. Invasive alien trees can also increase fuel loads and thus wildfire hazard [15], or can turn an ecosystem that was not prone to burning into a flammable landscape [16].

This growing body of evidence that land cover change influences the frequency and severity of natural hazards presents new opportunities for managing and reducing risks faced by society, and forms the foundation of ecosystem-based approaches to adaptation. These approaches seek to manage, conserve or restore ecosystems and their associated ecosystem services to help people cope with the impacts of climate change [17]. Ecosystem-based adaptation approaches can be used to complement or replace technological or engineering solutions to adaptation, and often present more cost-effective, self-sustaining and flexible alternatives in the long term [18], [19], [20]. Apart from enhancing the buffering capacity of ecosystems, these approaches often come with multiple co-benefits to humans, such as improved fisheries production [21], timber harvesting [22], biodiversity conservation [23], and recreational value [24].

A key challenge to incorporating ecosystem-based approaches into disaster risk reduction is quantifying the extent to which land cover changes influence the occurrence and consequences of natural hazards. Clear examples are needed to quantify the benefits of ecosystem-based approaches to disaster risk reduction. Such assessments will require, *inter alia*, understanding the multiple land cover and climate drivers of natural hazards, quantifying how ecosystems will respond to changes in these drivers, and examining trade-offs between ecosystem-based management relative to alternative solutions [19]. This paper describes a ‘proof of concept’ study which aimed to address the first two of these challenges, exploring how land cover and climate change might affect four natural hazards – floods, droughts, wildfires and storm-waves – in the south coastal region of South Africa. These natural hazards frequently affect southern Africa’s emerging economies and vulnerable communities. We limit the scope of this paper to natural hazards, but in the discussion reflect on translating natural hazards to disaster risk, which is the product of the likelihood of a natural hazard event occurring and its consequence on society. The intention of this proof of concept was to inform local authorities and businesses about local land cover drivers in their region and their potential for reducing the risk posed by natural hazards, thus contributing to comprehensive strategies for disaster risk reduction and climate change adaptation. But beyond informing local and national stakeholders in South Africa, the methods and lessons developed in this study have broader implications for ecosystem-based approaches and their evidence-base globally [19].

## Methods

### Study Area

**Figure 1:**
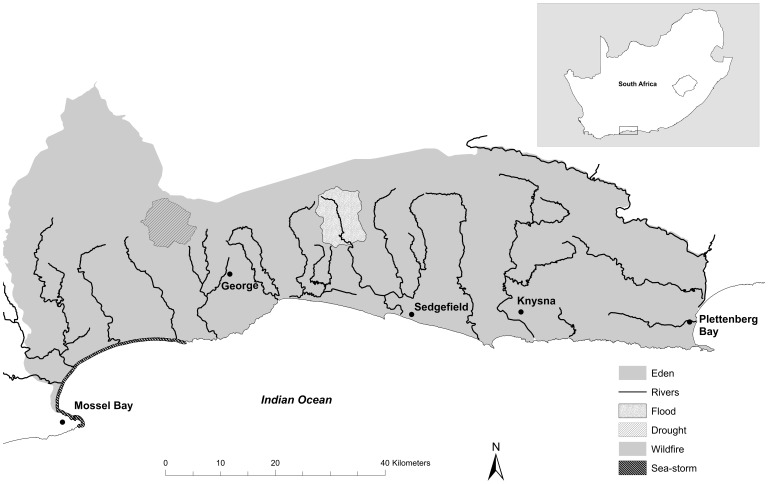
The location of the hazard model study areas within Eden. The inset showing the location of Eden in South Africa.

The study area, hereafter ‘Eden’ (Figure 1), is 3 820 km^2^ in size and comprises the local authorities along the southern Cape coast of South Africa within the Eden District Municipality (viz. Mossel Bay, George, Knysna and Bitou municipalities). Eden regularly experiences extremely heavy rainfall events which, combined with the small, steep catchments in the coastal areas, often results in high runoff and flash flooding [25]. The most severe incidences of flooding generally coincide with large storm-waves associated with cut-off low atmospheric pressure systems over southern Africa [26]. Floods are also interspersed with prolonged periods of extremely low rainfall, and Eden is frequently declared a disaster area due to persistent drought conditions [27]. The area also naturally experiences moderately frequent fires (every 10–13 years) due to the co-occurrence of its indigenous, flammable vegetation (‘fynbos’), periods of hot, dry weather, and readily available sources of ignition [28]. Intense wildfires pose significant risks when associated with high population densities, and the intensity of wildfires is further exacerbated by invasive alien trees, which increase the fuel loads [29], [30]. Natural hazards in Eden coincide with diverse socio-economic contexts, which results in inequalities to prepare for, cope with and adapt to disasters. Direct damage costs between 2003 and 2008 were estimated to be more than 3.5 times higher than the average annual household income in the most vulnerable and exposed communities, providing an indication of the vulnerability of some resident communities [25].

Approximately 68% of Eden’s surface area is covered by indigenous vegetation, with agriculture (lucerne, vegetables and hops) and timber plantations respectively comprising 17% and 12% of Eden [31]. Urban areas are concentrated along the coast, and estimated to occupy just over 2% of the area. Eden has a 26% population growth rate [32], which is well above the national growth statistic of 15%, and which places considerable pressure on both natural resources and built infrastructure in the region [27]. Almost all the remaining indigenous fynbos (98%) is invaded by alien plants to some extent, which translates to approximately 12% of Eden being invaded at 100% density [31].

Climatically, Eden is located in a transition zone between a winter and summer rainfall regime [33]. Rainfall occurs throughout the year, peaking in autumn (March) and spring (October) with the lowest monthly rainfall occurring in June. The spring and autumn rainfall peaks coincide with increased frequency of cut-off low pressure systems over southern Africa [26], and about one of every five of these brings flooding and damage to the coastal areas [34]. Being a transition zone makes the area particularly susceptible to climate change, because the climate is influenced by changes in the circulation systems of both climatic regimes. An increase in annual maximum temperatures of about 1.2°C has been observed since 1960 [35]. While trends in projected future annual rainfall are weak relative to temperature [36], the combined effects of temperature, rainfall and evapotranspiration are amplified in the hydrological cycle and can have profound impacts on water resources in southern Africa [37].

### Climate Data and Natural Hazard Models

Four models were developed to examine how land cover and climate change influence the natural hazards of floods, droughts, wildfires and storm-waves in Eden. Particular sites and catchments within Eden were selected to facilitate the modelling of each hazard (Figure 1). We identified drivers of each hazard based on literature reviews and expert consultations, highlighting those which were identified as important, relevant, plausible, and for which adequate data were available. The climate change scenario was extracted from the A2 scenario of the IPCC Special Report on Emission Scenarios (SRES) [38], while land cover change scenarios were based on published information and expert knowledge of the region. We then modelled the effect of the scenarios of change on each natural hazard (Table 1; Table S1). To explore the changes in flood, drought and storm-wave hazards, we produced simulations that respectively estimated return periods of extreme peak flow events, low flows and wave run-up events. The change in wildfire hazard was examined by calculating the change in fire intensities with future changes in fuel loads and climate.

**Table 1:** Scenarios of land cover and climate change used to quantify changes to flood, drought, wildfire and storm-wave hazards.

Hazard	Scenario
Flood	1. BASELINE: current land cover and climate
Flood	2. Clear-felling and non-replanting of the plantation; current climate
Flood	3. Burning down of the plantation by a moderate wildfire with replacement by degraded fynbos; current climate
Flood	4. Burning down of the plantation by a severe wildfire with replacement by degraded fynbos; current climate
Flood	5. Current land cover; future climate
Drought	6. BASELINE: Indigenous natural vegetation with no invasive alien plants or human activities; current climate
Drought	7. Alien trees invade to maximum potential; current climate
Drought	8. Indigenous natural vegetation with no invasive alien plants or human activities; future climate
Wildfire	9. BASELINE: Current levels of invasion by alien trees; current climate
Wildfire	10. Alien trees invade to maximum potential; current climate
Wildfire	11. Current levels of invasion by alien trees; future climate
Wildfire	12. Alien trees invade to maximum potential; future climate
Wildfire	13. Alien trees and shrubs are cleared and maintained at levels below 5% cover; future climate
Storm-wave	14. BASELINE: Current beach slope; current climate
Storm-wave	15. 3° increase in beach slope; current climate
Storm-wave	16. Current beach slope; future climate

More detailed descriptions of each scenario and the associated data used are available in Table S1.

#### Climate projections

Projections of future climate, used by the flood, drought and wildfire models, were obtained using a regional climate model, the conformal-cubic atmospheric model (CCAM) [39]. A multiple downscaling procedure was followed to obtain high resolution simulations of future climate change over southern Africa. In the first phase of the downscaling procedure, the sea-ice and bias-corrected sea-surface temperatures of the CSIRO Mark3.5 global climate model [3] was used as lower-boundary forcing in CCAM simulations performed globally at a quasi-uniform resolution of approximately 200 km [3], [40]. The simulations used the SRES A2 scenario [38], which was the only detailed downscaled data for the southern African region. The A2 scenario represents a low mitigation, or high emissions, scenario implying rapid continued growth in greenhouse gas concentrations in the atmosphere. It is thus appropriate in examining the impacts of extreme climate events, although we do note throughout the limitations of only having one climate scenario available. CCAM was subsequently applied in a stretched-grid mode, to obtain simulations of approximately 60 km resolution in the horizontal, over southern Africa. This CCAM modelling procedure has been shown to provide satisfactory simulations of annual rainfall and temperature distributions, as well as the intra-annual cycle in rainfall and circulation over the southern African region [40]. It also realistically simulates observed daily climate statistics over southern Africa, such as the frequency of occurrence of extreme precipitation events, and the cut-off low atmospheric pressure systems and tropical cyclones [40], [41]. Climate data required for each natural hazard model were extracted for the period 1961–2050, which included daily data for: rainfall, maximum temperature, wind speed, and relative humidity (Dataset S1).

#### Modelling flood hazard

We developed a hydrological model for an upper catchment within Eden (Figure 1) that drains into a coastal lake near the low-lying town of Sedgefield, which has in the past been highly susceptible to flooding during heavy rains. The model explored how inflows to the lake are affected by catchment land cover and climate change. This upper catchment was chosen because it had reliable gauging weir data for calibrating simulated flows, and included the typical land cover found in Eden.

The agrohydrological modelling system (ACRU) was used to simulate daily flows based on climate data, physical catchment characteristics (particularly soils) and land cover [42]. ACRU contains a soils and vegetation database that was developed specifically for South African conditions, and has been widely used, both locally and elsewhere [43], [44]. We used this database, together with 30 m resolution land cover data [45], to identify five ‘hydrological response units’: pine plantation, clear-felled pine plantation, wattle plantation, indigenous forest and fynbos. We constructed a model for baseline conditions using daily temperature for 1961–1990 from our climate model, and daily rainfall [46] for the same period (Table 1). The latter was considered to better reflect the rainfall gradients within the catchment than the relatively coarse resolution of our climate model. All hydrological response units fed directly into the outflow point at the gauging weir, so that simulated flows could be calibrated against observed flows (Table 1). The initial settings in the ACRU model were based on default values in the model for land cover classes and soils [47]. The simulated flows were much greater than the observed flows and too responsive to rainfall events so: (i) soil and effective rooting depths were increased to accommodate regional variations in soils and greater rooting depths for fynbos and pine trees [43]; and (ii) quickflow and baseflow response coefficients were altered according to Royappen et al. [48] for this catchment. These adjustments brought the mean simulated flows to within 10% of the observed daily flows, which were used to describe the baseline for runoff events.

We used this calibrated model to explore the implications of different forestry management practices and climate change on flood events (Table 1). Future daily temperature data for 2021–2050 were extracted from our climate model. To account for the inadequate rainfall resolution, future daily rainfall was calculated using a proportional adjustment to the current daily rainfall data [46], based on regional trends in current and future daily rainfall from the climate model used here. Regional trends were calculated as proportional differences for modelled daily rainfall percentiles and then applied to the corresponding percentiles of the observed data to generate the future rainfall for 1991–2020 and 2021–2050. The exceedance probabilities for extreme flows for both current (1961–1990) and future (2021–2050) climate conditions were calculated from the simulated flow record using the Log Pearson III distribution, which is widely used for calculating extreme values and their return intervals [49]. An extreme rainfall event in the climate model dataset was defined as 25 mm of rain falling within 24 hours over a grid cell of 0.5° latitude**×**0.5° longitude.

#### Modelling drought hazard

Many definitions of drought exist – e.g. ‘meteorological’, ‘atmospheric’, ‘hydrological’, ‘agricultural’ and ‘water management’ droughts [50] – each differing in their emphasis on describing the characteristics and causes of drought, vulnerability to drought, or impacts of drought. Here, we study the effects of land cover and climate on hydrological drought, which is defined as a persistently low discharge or volume of water in streams or reservoirs, lasting months or years. Although hydrological drought is a natural phenomenon underpinned by dry climate, it is greatly exacerbated by human land use activities, which often affect the magnitude and frequency of the drought [50]. We used flow duration curves to explore changes in hydrological patterns. Flow duration curves plot the percentage of time a flow exceeds a certain threshold, with flows between 70–99% exceedance depicting low flows [51], and flows with >90% exceedance describing extreme low flows and taken here to represent drought.

Flow simulations used to develop flow duration curves were derived for a headwater sub-catchment within Eden (Figure 1) using the Pitman model [52], [53], [54]. This sub-catchment supplies water for high-value hops farming, and inadequate flow, particularly during the dry season, is frequently problematic [27]. The Pitman model is a conceptual monthly time-step rainfall-runoff model that has been frequently used for water resource assessments in southern Africa for many years and has become a standard method used by many practitioners. It includes explicit routines to simulate interception, infiltration, excess surface runoff, soil moisture (or unsaturated zone) runoff, groundwater recharge and drainage to stream flow, as well evaporative losses from the unsaturated zone and the groundwater storage in the vicinity of the river channel. We used a physically-based parameter estimation procedure described previously [55], [56], which uses physical property data at the sub-basin scale (typically 50–1000 km^2^). The agricultural land types GIS layer for South Africa [57] provides much of the physical property data required, including soil depths for different parts of the sub-basin (hilltop, valley sides and valley bottoms), soil texture (translated into soil hydraulic properties), topographic slope, and sub-surface geological conditions. Five land types were identified in the study area (Db30, Db32, Fc42, Lb139 and Lb141), based on spatial land cover data [45], and these were used to estimate runoff generating, soil moisture accounting and groundwater parameters for the Pitman model. The model was then run to generate time-series of mean monthly runoff under baseline conditions (Table 1). Resulting output ensembles were within the 90% confidence intervals of the regionalised mean annual runoff ratios, the gradient of the monthly flow duration curve, and within range of the three recharge estimates of the groundwater resource assessment study [58].

Using the calibrated Pitman model, we then explored changes in flow resulting from maximum potential invasion by alien trees and from climate change (Table 1). An ecological module was used to estimate invasive alien tree water use [59], which links back to the Pitman model to predict changes in streamflow [60]. Alien trees were only allowed to invade untransformed vegetation, which were calculated as those areas not classified as urban, agriculture, forestry plantation or waterbodies in the land cover GIS layer for South Africa [45]. We used monthly temperature and rainfall data from our climate model. The inadequate resolution of the rainfall data was addressed by scaling the projected future rainfall according to the monthly distribution statistics of historical rainfall data as outlined previously [61], [62]. Changes in flow for each scenario were compared using flow duration curves, focusing on flows produced at >90% exceedance range.

#### Modelling wildfire hazard

The damage caused by a wildfire is directly related to Byram’s fireline intensity [63], which measures the rate of energy released along the fire front. The relative effort required to control a fire, and the damage it does, are both strongly correlated with fireline intensity [64]. The higher the fireline intensity, the more difficult a fire is to control, which has important implications for human safety and fire-sensitive assets [65]. Wildfires are a regular occurrence in the fire-prone indigenous fynbos in Eden [28]. However, invasion by alien trees have the effect of increasing the above-ground biomass or fuel, and therefore fire intensity [29]. If uncontrolled, invasive alien trees will continue to spread leading to further increases in fire intensity. In addition, climate change is predicted to result in hotter, drier and windier weather, which will further increase the intensity of wildfires.

We used Byram’s [63] equation (Equation 1) to estimate changes in fireline intensity across 106 spatial assessment units in Eden under different scenarios. These assessment units are similar-sized irregular areas (mean size = 49 km^2^) that are nested within administrative and physiographic boundaries [66].
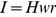
(1)Where I = fireline intensity in kW m^–1^;

H = heat yield (assumed to be constant at 20000 J g^–1^);

w = fuel loads in g m^–2^; and

r = rate of fire spread in m s^–1^

For each assessment unit, we assumed that the area covered by untransformed fynbos vegetation (as calculated for the drought model) would be available to burn in wildfires. We considered five scenarios, each with a unique combination of climate and invasive alien trees (Table 1).

We used recent spatial data [31] on the area invaded by pine trees and hakea shrubs (*Pinus* and *Hakea* species) to estimate fuel loads in each assessment unit under different scenarios. Pines have annual spread rates of 3.75–20.6% in the fynbos biome [67], [68], [69], and hakea can spread at 8% annually [70]. We assumed that pines and hakea would spread at a conservative rate of 4% per year into the available area within each assessment unit (to a maximum of 100% of untransformed vegetation only), and used this to estimate the area that would become invaded by 2050.

Fuel loads in fynbos vegetation are approximately 1 800 g m^−2^ [29]. Invasion by hakea and pines can increase these fuel loads to 3 900, and to 20 000 g m^−2^, respectively [29], [30]. The proportional mix of invasive species in Eden as a whole was 72.5% pine and 27.5% hakea, so we assumed a mean fuel load for assessment units of 15 572 g m^−2^. Based on these biomass estimates, we used the relative proportions occupied by uninvaded and invaded fynbos respectively to estimate a mean fuel load for each assessment unit under the different scenarios.

We used data on fire weather conditions and associated rates of fire spread in fynbos [71] to establish the relationships between McArthur’s Forest Fire Danger Index (FDI) [72] and rates of fire spread (Equation 2). McArthur’s FDI is based on observed relationships between the behaviour of fires and the environmental conditions (air temperature, relative humidity and wind speed) under which they burn. The FDI provides an index of the degree of difficulty of suppressing a fire.
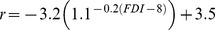
(2)Where r = rate of fire spread in m s^–1^ and FDI = McArthur’s Forest Fire Danger Index

Equation 2 was used to convert FDIs to estimates of current (1961–1990) and future (2011–2050) rates of fire spread using daily data from our climate model for temperature, wind speed, rainfall, and relative humidity. We then used the resultant mean rates of spread for current and future conditions (1.1 m s^–1^ and 1.2 m s^–1^ respectively) to estimate the fire intensity under different scenarios (Table 1).

#### Modelling storm-wave hazard

Storm-waves, in the southern African context, refer to extreme offshore wave events which result in wave impacts that are experienced at the shoreline. The severity of storm-wave impacts is highly dependent on wave run-up height (the maximum point that storm waves can reach on land) and coastal erosion potential [73]. Two models were developed to examine the spatial and temporal variation of wave run-up and coastal erosion potential within the study area and how these are likely to change in the future.

First, we used a numerical wave model, SWAN [74], to translate offshore wave data to inshore wave conditions. Offshore data on wave height, period and direction, from the National Centre for Environmental Prediction (NCEP), were used as input variables [75], [76]. Simulations were run to determine inshore wave heights for various return periods. Simulations included offshore wave conditions that result from 1∶10 year south-south-westerly swells, as these conditions result in severe inshore conditions in the study area. Second, we used the inshore wave height and period from the SWAN wave model simulations as input into the Nielsen and Hanslow model [77] to calculate wave run-up elevations at 0.5 km points along the coastline, together with corresponding return periods. This model requires information on inshore wave height and period, sea water level and beach slope, and has previously shown an acceptable prediction accuracy (R^2^ = 0.79) for local South African conditions [73]. We used wave heights from the SWAN model simulations as inputs for the inshore wave height and period, and sea water level was based on spring high tidal level predictions. This is a relatively extreme but realistic scenario as spring high tides occur every 14 days along the coastline, making the chances of storm waves coinciding with spring high tides relatively high. Beach and inshore slopes were calculated using the distance to the 20 m depth contour obtained from the South African Navy’s bathymetric charts and available 5 m contour intervals. Using these outputs, baseline conditions of wave run-up elevations and corresponding return periods were plotted for each 0.5 km location along the coast (Table 1; Figure 1).

These models were then used to examine the change in wave run-up elevations resulting from the future potential influence of anthropogenic effects on coastal erosion (resulting in a steeper slope), and the change in future wave climate and sea-level rise (Table 1). Anthropogenic effects on coastal erosion were simulated by assuming a 3° increase in beach slope which was used as input into the Nielsen and Hanslow run-up model [77]. This is a conservative increase in beach slope that local coastal engineers considered to be a realistic effect of coastal erosion, which occurs as a consequence of human activities, e.g. hardening of the coastline through urban and industrial development, removal of coastal foredunes. Future climate conditions were modelled by assuming a 0.5 m rise in sea-level based on reviews of recent publications [78], [79], and by applying a 6% increase to offshore extreme waves based on regional projections from metocean climate modelling [80]. We calculated expected changes in wave run-up elevation and return period up to the year 2100 and compared these to baseline conditions.

## Results

**Figure 2:**
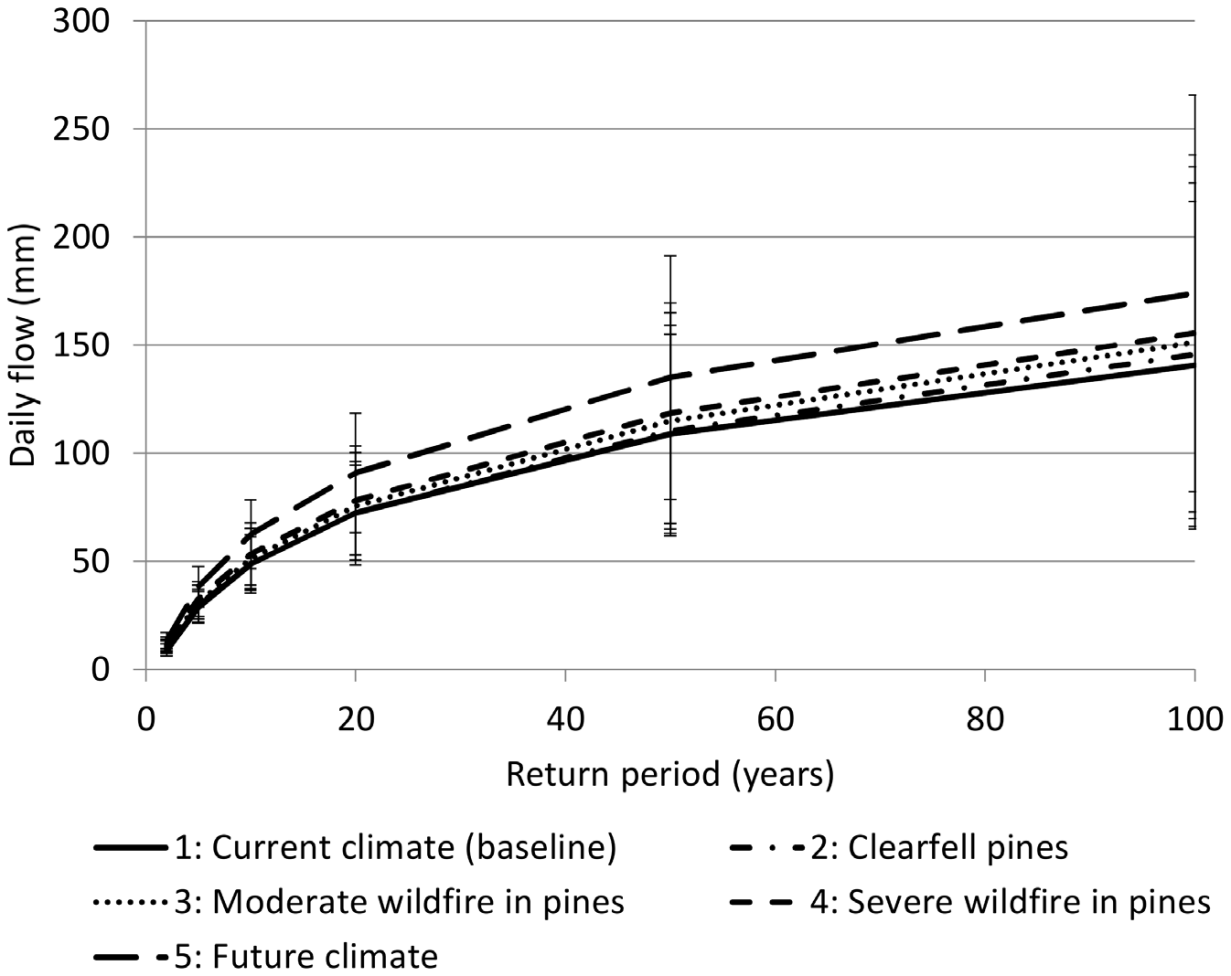
Flood return intervals for different scenarios of land cover and climate change. The numbers prefixing the annotated description of each scenario provides a reference to Table 1, which describes each scenario in more detail. The changes in the values for each return interval illustrate the potential changes in the likelihood of extreme flow events under the different scenarios. For example, the return period of a flood with a daily flow of 150 mm (similar to the May 1981 flood in this area) would decrease from a baseline of more than 100 years to 70 years under future climate (scenario 5).

The flood model for all land cover and future A2 climate scenarios showed an increase in extreme daily flows for equivalent return periods compared to the current baseline condition (Figure 2). Estimated extreme daily flows for a two-year return period showed a 16% increase from the baseline condition for the scenario of clear-felling without replanting. Similarly, the scenarios describing the burning down of the timber plantation under moderate and severe fires show an increase of approximately 24% and 32% respectively. The A2 scenario of future climate change also shows an increased trend in extreme daily flows, estimated to increase by over 24% of the baseline for the 50-year return period. Under the A2 scenario of climate change, flood return intervals are substantially reduced, doubling the frequency of 1∶100 year floods to 1∶50 years. Land cover changes also resulted in increases in flood frequency, shifting the 1∶100 year flood event to about a 1∶80 year return period in the most extreme scenario in which timber plantations were burnt down by a severe fire and replaced with degraded fynbos (Figure 2).

**Figure 3:**
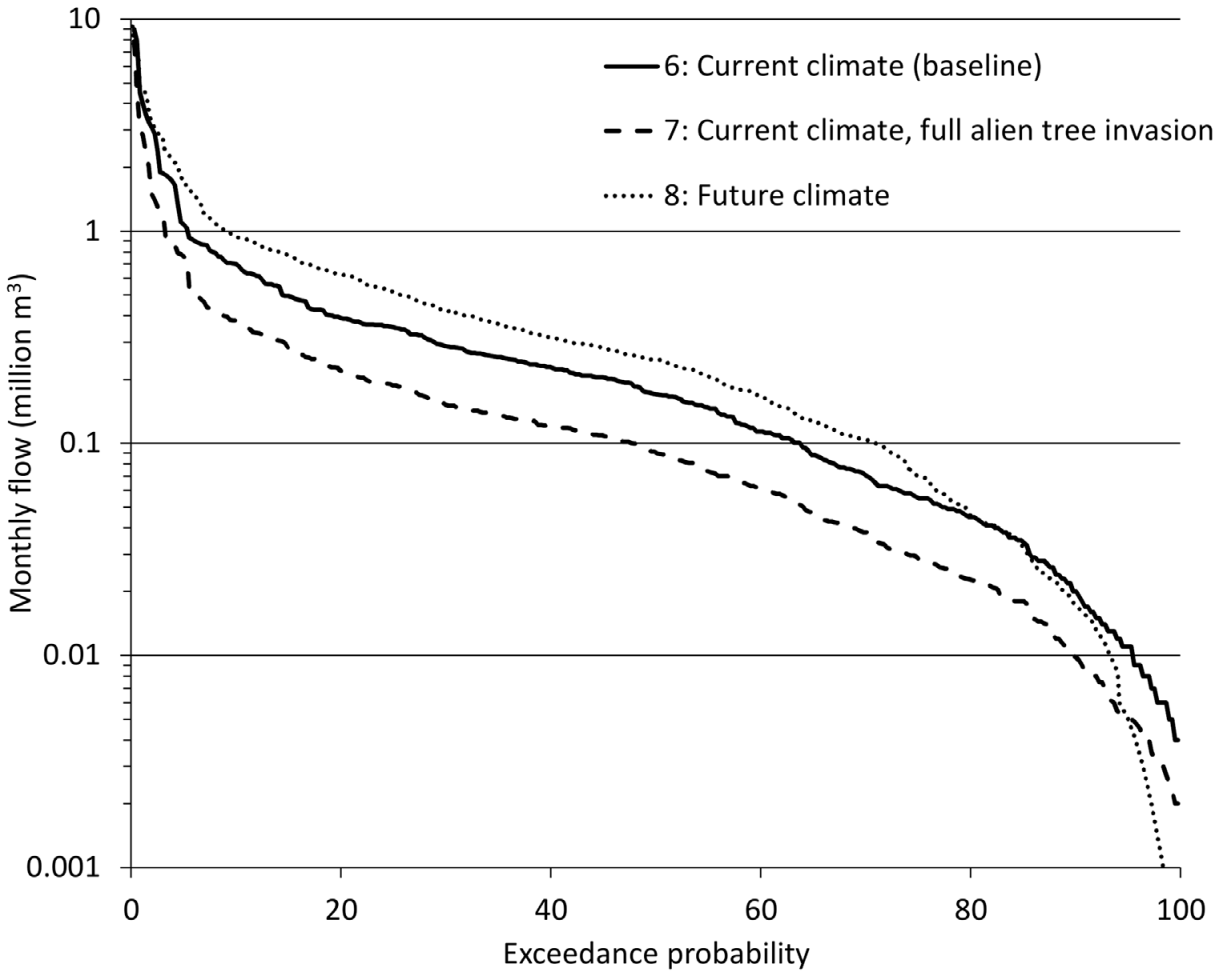
Flow duration curve for different scenarios of land cover and climate change. This shows the cumulative proportion of the months where a flow exceeded a given discharge for the different scenarios. The numbers prefixing the annotated description of each scenario provides a reference to Table 1, which describes each scenario in more detail. Extreme low flows were defined as those with >90% exceedance, which were used in this study to represent severe drought conditions. A log-normal probability curve was used to allow the low and high flow ends of the plot to be more clearly displayed.

The full range of river discharges in the drought study is displayed in Figure 3, from the low flows to the high flows (broadly defined as flows respectively above or below 50% exceedance). Figure 3 shows that both invasion by alien trees and future climate change (under the A2 scenario) will exacerbate extreme low flows (flows >90% exceedance). Flow for alien trees at maximum potential invasion were consistently lower than baseline flows, halving the expected flow at 90% exceedance (0.02 million m^3^ under baseline compared to 0.01 million m^3^ under full invasion; Figure 3). While the A2 climate change scenario shows an overall trend of higher flows, its flow duration curve drops sharply below the baseline at 90% exceedance, suggesting that under this scenario of climate change much lower flows will be experienced during drought periods.

**Figure 4:**
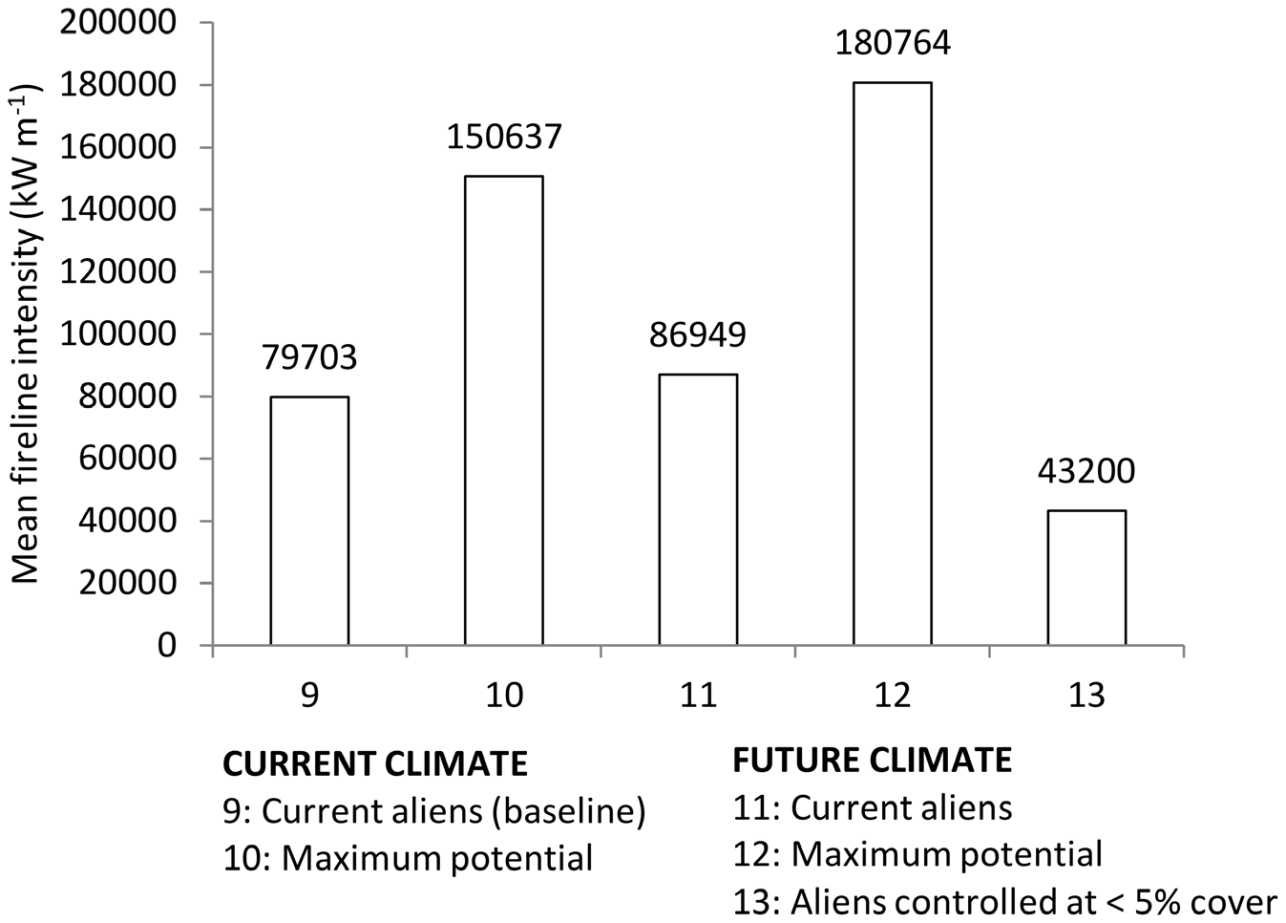
Estimated mean fireline intensity experienced in 106 assessment units for different scenarios of climate change and alien tree management. The numbers prefixing the annotated description of each scenario provides a reference to Table 1, which describes each scenario in more detail.

Mean fireline intensities of around 80 000 kW m^–1^ were estimated under current baseline conditions (Figure 4). This could increase by about 88% (to 150 000 kW m^–1^) under a scenario in which invasive alien trees continue to spread unhindered, without any climate change, and could potentially more than double (to over 180 000 kW m^–1^) if the combined effects of alien tree spread and the A2 scenario of climate change are taken into account. Under a hypothetical scenario in which climate change took place, but alien tree invasions remained at current levels, mean fireline intensities would increase by about 9% (to 87 000 kW m^–1^) under the A2 scenario of climate change compared to current conditions. However, under a scenario in which alien trees are brought under control by reducing and maintaining them at below 5% cover, the estimated mean intensity of fires would be reduced by almost half (to 43 200 kW m^–1^) when compared to the current situation.

**Figure 5:**
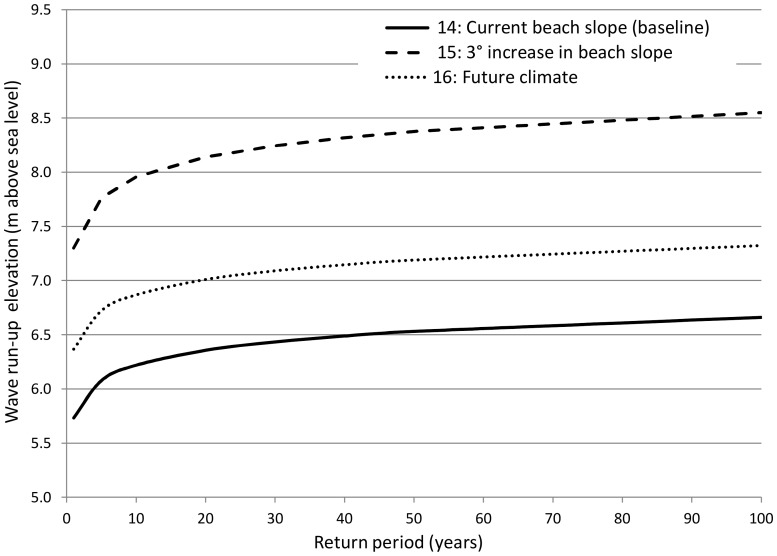
Wave run-up elevations for various storm-wave return intervals for different scenarios of beach slope and climate change. Simulations used here are for a typical sandy beach location in Eden (Tergniet, near Mossel Bay), which is prone to storm-wave damage. Return periods were based on the simulated wave run-up elevations for a south-south westerly swell, and spring high tide levels. The numbers prefixing the annotated description of each scenario provides a reference to Table 1, which describes each scenario in more detail.

Using a sandy beach location as an example of a typical area prone to storm-waves in Eden, simulations of wave run-up elevation for spring high tide and south-south-westerly swell conditions are currently predicted to range between approximately 5.7 m for a 1∶1 year return period to 6.5 m for a relatively extreme event with a 1∶50 year return (Figure 5). Under future wave climate and sea-level rise predictions, the 1∶50 year wave run-up elevation will be reduced to a 1∶3 year return interval at this location. Increasing beach slope by 3° to simulate conservative anthropogenic effects on coastal erosion, produced a substantial increase in wave run-up elevation for respective return periods. In this scenario, the wave heights of a 1∶100 year return period are likely to occur on an annual basis.

## Discussion

### Natural Hazards are Increasing

Our results show that climate change in Eden will increase the frequency of all natural hazard events examined, substantially so in the case of floods, droughts and storm-waves (Figures 2–5), and to a lesser extent for wildfires (Figure 4). When looking beyond climate change impacts, drivers of land cover change appear to have a similar effect on increasing the incidence of natural hazards (Figures 2–5). Allowing the spread of invasive alien trees into untransformed vegetation was estimated to halve the monthly river flows experienced during drought and double fire-line intensities (Figures 3 and 4 respectively). In the case of wildfire (Figure 4), the fireline intensities for invaded fynbos are all orders of magnitude greater than the limits for effective fire control or suppression [81]. This poses significant threats to life and infrastructure [65], and can have significant hydrological impacts [82]. Similarly, severe 1∶100 year storm-waves are estimated to occur on an annual basis with only modest human-induced coastal hardening and the removal of coastal foredunes (Figure 5). The impacts of land cover change on floods also attest to the impacts of land management practices on natural hazards by reducing the return time between large flood events by nearly 20% (Figure 2).

In interpreting these findings, we focus on broad trends rather than quantitative measurements because of the inherent uncertainty, gaps in data and models, and limited scenarios explored in predicting land cover and climate change impacts. With only one climate change scenario (focusing on a high emission, high risk scenario), we cannot compare land cover and climate change impacts with great certainty. However, we can point out that even under this extreme climate change scenario, land cover changes were shown to have as great and sometimes greater impacts on natural hazards, highlighting the importance of land cover management in reducing natural hazards. When considering the land cover scenarios, our focus was usually on single drivers of land cover change (e.g. spread of alien trees or forestry management practices); however, even within this small set of land cover drivers, the impacts were substantial on natural hazard incidence. As more climate scenarios become available for southern Africa, as well as data on other land cover and land use drivers relevant to natural hazards (e.g. agricultural practices, ground water abstraction), the additional impacts of these on natural hazards can be incorporated into our models and integrated into other ecosystem-based management options. In moving beyond this study’s focus on general trends and evidence of importance of land cover and climate change drivers on natural hazards, future work on new drivers, data and scenarios would help in better reflecting the ranges of impacts, and the uncertainty associated with the predictions. This in turn will improve the ability to examine trade-offs of alternative solutions to disaster risk reduction and climate change adaptation.

Data and modelling techniques are also continuously evolving, and several improvements can be made to increase the confidence of the hazard models. For example, within specific natural hazard models (e.g. wildfire) the limited availability of fine scale data resulted in the use of a relatively coarse grid scale, possibly under-estimating the impacts found. An analysis of extremes (rather than means) at a finer resolution may show higher climate-related change (see Figure S1). Moreover, models that explore interactions between drivers and between natural hazards would be more informative than the separate models produced here. For example, droughts and wildfire are closely linked hazards with similar climate drivers (warmer and drier conditions). Droughts can exacerbate wildfires because they usually increase dry, highly flammable standing biomass [6]. Severe wildfires also exacerbate floods, especially when they precede the onset of rains. This is particularly the case when wildfires occur in densely invaded areas, which burn more intensely than indigenous vegetation, and which result in increased runoff caused by resin-induced water-repellency and associated reductions in infiltration [84].

### Natural Hazards can be Reduced by Appropriate Land Management

This study makes a case for the incorporation of ecosystem-based management approaches, in tandem with other approaches (e.g. mitigation or engineering responses), into disaster risk reduction and climate change adaptation. Our findings show that through appropriate and pro-active land use management, it would be possible to reduce the impacts of natural hazards to a large degree. Clear-felling of timber plantations should ideally be associated with rehabilitation and re-vegetation to avoid increasing the flood hazard. Because the timber plantations (mostly *Pinus* sp) are similar to dense stands of invasive alien trees, the flood model also supports the clearing of invasive alien trees to reduce the hazard posed by flood events soon after a wildfire. Clearing invasive alien trees and restoring the natural fynbos vegetation is also an effective tool for reducing wildfire and drought hazards. The wildfire model shows that the impacts of climate change can be substantially reduced by clearing invaded areas and maintaining a healthy cover of indigenous vegetation, lowering the fireline intensity to half that of current levels (Figure 4).

The costs to clear existing invasive alien trees in Eden are much smaller than the estimated losses caused by damaging wildfires to, for example, timber plantations. Estimates of the cost of clearing of invasive trees range from US$ 100 ha^−1^ [85] to US$ 800 ha^−1^ [86], giving estimates of between US$ 2.3–19.2 million to clear in the Eden area. A single wildfire in 2007 caused losses of US$ 200 million to the local timber industry [87]. Similarly, estimated costs of clearing were estimated to be lower than the economic impact invasive alien trees have on the hops industry in Eden (c.a. US$ 250 000 per year for 15 years, compared to US$ 350 000 per year for perpetuity to cover the additional groundwater pumping costs) [88]. Similar invasive alien tree clearing initiatives have been proposed to lower the long term economic impacts of natural hazards to the fruit industry in Eden [27].

Human activities that harden the coastline exacerbate beach erosion, thereby increasing beach slope and wave run-up [8]. Coastal foredunes are the South African equivalent of salt marsh and mangrove wetlands that offer protection from hurricane storm-surge or tsunamis elsewhere in the world [8], [21]. Maintaining natural vegetation, sand volume, and natural sediment movement, and restricting developments on foredunes can reduce impacts of wave run-up. Given that human-induced coastal erosion substantially influences the impacts of extreme storm-wave events (Figure 5), rehabilitation of foredunes should also be seriously considered as a means of reducing the impacts of climate change. Indeed, this is the strategy that has seen the US government investing billions of dollars in restoring the coastal salt marshes to protect the Gulf of Mexico from extreme events such as Hurricane Katrina in 2005 [21], [89].

### Way Forward in Eden and in Ecosystem-based Adaptation

The broad trends from this ‘proof of concept’ study have provided sufficient evidence to mobilise action within Eden, and several public-private initiatives have been launched to clear invasive alien trees, and restore catchments and foredunes. Implementation mechanisms have also been established to facilitate local action, including the appointment of a catchment manager by the hops farming industry, and the establishment of the ‘Business Adopt a Municipality’ forum in Eden, which explores how best the insurance sector can support local authorities and communities to manage their natural hazards and environmental risk.

While refining the hazard models is clearly important in identifying on the ground actions, perhaps more important is the need to understand how these natural hazards and associated trends express themselves as risk to communities in Eden, so that they can prepare for, cope with, and recover from disasters [90], [91], [92]. Disaster risk is widely accepted as the product of a natural hazard and its consequences on society [93]. The latter depends on the exposure and vulnerability of communities to natural hazards [3]. Eden has a naturally high relative exposure, and this coincides with particularly vulnerable communities living in the area [25]. Farmers are highly affected by repeated setbacks from droughts, floods and wildfires, and this has severe ripple effects on rural farm labourers and the entire local economy. This, in turn, increases the vulnerability of communities in Eden, making them more susceptible to subsequent disasters.

Disaster risk reduction is an activity that seeks systemic ways to reduce the severity or occurrence of natural hazards, as well as the consequences that such events have on people [93]. Eden has a relatively well-capacitated Disaster Risk Reduction unit compared to other districts in South Africa. However, much like in many parts of the world, efforts are still very much focussed on recovery from disaster (e.g. through providing disaster relief funding), or short-term disaster preparedness (e.g. through early warning systems, or ensuring adequate supply of fire engines). Longer term efforts to reduce risk are still lacking. These could include efforts to manage drivers of risk through appropriate land use management and restoration, as well as providing opportunities for social learning that promotes individual, collective and institutional capacity to manage risk [91]. Although the disaster risk framework in South Africa (Disaster Management Act No. 57 of 2002), and more widely, acknowledges both short and longer term efforts, there is still a lack of explicit budget allocated to longer term efforts. This expresses itself at the local level where authorities, such as the Eden District Municipality, that are interested in piloting new approaches, have no funding to do so. Interventions at all levels of governance (local, provincial and national) will be required to remove financial implementation barriers.

## Conclusions

There is a need to build an evidence-base that addresses the potential of ecosystem-based adaptation approaches [19]. Our models and findings contribute to such a call. Our study shows that land cover change is as important as climate change in influencing the effects of natural hazards. These findings offer the Eden community an empowering message. Through pro-active management of key drivers of land cover change, they will be able to reduce the impacts of floods, droughts, wildfires and storm-waves. In considering the trade-offs of such ecosystem-based approaches with alternative forms of disaster risk reduction, the multiple co-benefits of ecosystem management and restoration need to be considered. For example, clearing invasive alien trees will reduce the impacts of drought, wildfire and flood hazards, create opportunities for employment in rural poor communities, and decrease the vulnerability of agricultural production and thus the overall local economy of the region. Promoting individual, collective and institutional capacity to deal with risk through social learning networks is also increasingly being recognised as an important long term strategy in disaster risk reduction [91], [92]. Indeed, a recent study found that socio-institutional interventions tended to offer the most efficient climate change adaptation options in the city of Durban, South Africa [94]. The establishment of the public-private initiatives between land-owners, businesses and government agencies in Eden is a good step in this direction, as it offers the opportunity for cross-sectoral collaboration and social learning around disaster risk reduction.

## Supporting Information

Figure S1 **Fire Danger Index (FDI) calculated for the current (1960–1990) and future (2010–2050) time periods using the CCAM climate data for temperature, wind speed, rainfall, and relative humidity.** Although there is a strong directional change in temperature, this trend is mediated by relatively small changes to wind speed and relative humidity. However, the small change to wind speed and relative humidity may be the result of an averaging effect, because both parameters are highly variable within the relatively coarse grid cells of the CCAM climate model. An analysis of extremes (rather than means) within the grid cell may show higher climate-related change.
(TIF)

Table S1 **Scenarios of land cover and climate change used to quantify changes to flood, drought, wildfire and storm-wave hazards, together with the associated data used in each scenario.**
(DOCX)

Dataset S1 **Climate model data used in the natural hazard models for the period 1961–2050, which included daily data for rainfall in mm over a 0.5° latitude×0.5° longitude grid cell (Rain), maximum temperature in °C (MaxT), wind speed in km/h (Wind), and percentage relative humidity (RH).**
(XLSX)
