# Supplementary figures and images for: Natural Hazards in a Changing World: A Case for Ecosystem-Based Management
Source: PLoS One. 2014 May 7;9(5):e95942. doi: 10.1371/journal.pone.0095942 (PMC4012988; doi:10.1371/journal.pone.0095942)

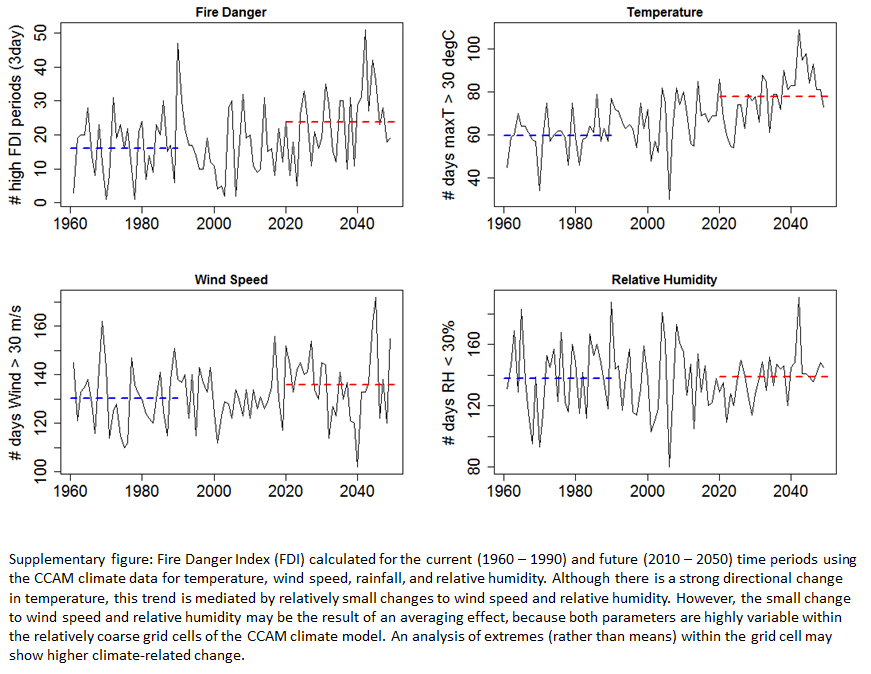

Supplement: Figure S1 — Fire Danger Index (FDI) calculated for the current (1960–1990) and future (2010–2050) time periods using the CCAM climate data for temperature, wind speed, rainfall, and relative humidity. Although there is a strong directional change in temperature, this trend is mediated by relatively small changes to wind speed and relative humidity. However, the small change to wind speed and relative humidity may be the result of an averaging effect, because both parameters are highly variable within the relatively coarse grid cells of the CCAM climate model. An analysis of extremes (rather than means) within the grid cell may show higher climate-related change. (TIF) [file pone.0095942.s001.tif]
