# Supplementary material for: Natural Hazards in a Changing World: A Case for Ecosystem-Based Management
Source: PLoS One. 2014 May 7;9(5):e95942. doi: 10.1371/journal.pone.0095942 (PMC4012988; doi:10.1371/journal.pone.0095942)
Supplement: Table S1 — Scenarios of land cover and climate change used to quantify changes to flood, drought, wildfire and storm-wave hazards, together with the associated data used in each scenario. (DOCX) [file pone.0095942.s002.docx]

Supplementary Table S1: Scenarios of land cover and climate change used to quantify changes to flood, drought, wildfire and storm-wave hazards, together with the associated data used in each scenario.

|  | **Scenario** | | | **Description and associated data** | |
| --- | --- | --- | --- | --- | --- |
| **Flood** | 1 | BASELINE: current land cover and climate | | Hydrological response units delineated using current 2000 national land cover (van den Berg et al. 2008). Daily temperature from our climate model for 1961-1990.Daily rainfall for the same period from Lynch et al. (2004). Observed flow gauge data from Department of Water Affairs flow gauge K4H003 (http://www.dwa.gov.za/Hydrology/RTData.aspx?Station=K4H003EC&Type=Data) | |
|  | 2 | Clear-felling and non-replanting of the plantation; current climate | | Non-replanting of plantations is frequent when not economically viable. The land recovers to a moderately degraded form of the original fynbos, resulting in decreased transpiration losses, adjustment of soil the moisture regime, and increased streamflow (Scott et al. 2000). To simulate these conditions, the 'Pine plantation' and 'Wattle plantation' hydrological response units in scenario 1, representing 25% of the sub-catchment area, were adjusted to reflect the same vegetation parameters as for 'Pine clear-felled'. Climate data as for scenario 1. | |
|  | 3 | Burning down of the plantation by a moderate wildfire with replacement by degraded fynbos; current climate | | Moderate wildfire causes vapourised resins from the plantation litter to aggregate on soil particles below the surface, which increases the water repellency of the soil, reducing rain infiltration rates (Scott 1993). The quick flow responsiveness of affected hydrological response units was increased to simulate reductions in rain water infiltration rates. Quantitative measures of quickflow responsiveness were based on comparing published findings for soil responsiveness in natural systems (Shakesby and Doerr 2006) to soil responsiveness after wildfire in mature stands of pine (van Wilgen and Richardson 1985). Climate data as for scenario 1. | |
|  | 4 | Burning down of the plantation by a severe wildfire with replacement by degraded fynbos; current climate | | Following severe wildfire, the water repellent layer forms slightly deeper in the soil compared to the moderate wildfire in scenario 3 and surface soil carbon is burnt, destroying the soil structure. Quickflow responsiveness is increased accordingly (see scenario 3). Climate data as for scenario 1. | |
|  | 5 | Current land cover; future climate | | Future daily temperature data from our climate model for 2021-2050. Observed daily rainfall from the baseline scenario was projected into the future based on regional trends in current and future rainfall from our climate models. Regional trends were calculated as proportional differences for different modelled daily rainfall percentiles and applied to the corresponding percentiles of the observed data (1961-1990) to generate the future rainfall (2021-2050). Current land cover as for scenarios 1-4. | |
| **Drought** | 6 | | BASELINE: Indigenous natural vegetation with no invasive alien plants or human activities; current climate | | Indigenous natural vegetation based on five land types according to the AGIS (2007) spatial database. This database was used to derive physical data (mainly soils) used to generate run-off, soil moisture accounting and groundwater parameters in the hydrological model. |
|  | 7 | | Alien trees invade to maximum potential; current climate | | Maximum, dense invasion of alien trees in untransformed vegetation, which are those areas not classified as urban, cultivated or waterbodies in van den Berg et al. (2008). Associated alien plant water use modelled using Le Maitre et al. (1996) ecological model and linked back to the Pitman hydrological model (Middleton and Bailey 2009). Climate data as for scenario 6. |
|  | 8 | | Indigenous natural vegetation with no invasive alien plants or human activities; future climate | | As for scenario 6, but with future climate. Future monthly rainfall and temperature from our climate models for 2021-2050. The inadequate resolution of the rainfall data was addressed by scaling the simulated rainfall from our climate model according to the monthly distribution statistics of historical rainfall data as outlined in Hughes et al. (2011). The rain time-series thus derived was downscaled to the study sub-catchment using an area-weighted and mean annual precipitation ratio (Hughes 2004). |

*contd*

|  | **Scenario** | | **Description** | |
| --- | --- | --- | --- | --- |
| **Wildfire** | 9 | BASELINE: Current levels of invasion by alien trees; current climate | | Current levels of invasion by alien trees based on Vroman et al. (2010). Levels of invasion were quantified as area-weighted densities (area occupied at 100% density). Daily temperature, relative humidity and wind speeds according to our climate model for 1961-1990. |
|  | 10 | Alien trees invade to maximum potential; current climate | | Invasive alien trees, as per Vroman et al. (2010), spread uncontrolled at a rate of 4% per year in all untransformed vegetation (i.e. areas not classified as urban, cultivated or waterbody as per van den Bergh et al (2008) land cover). Climate data as for scenario 9. |
|  | 11 | Current levels of invasion by alien ; future climate | | As for scenario 9 except with future daily temperature, relative humidity and wind speeds extracted from our climate model for 2011-2050. |
|  | 12 | Alien trees invade to maximum potential; future climate | | As for scenario 10 except with future climate according to scenario 11. |
|  | 13 | Alien trees and shrubs are cleared and maintained at levels below 5% cover; future climate | | Invasive alien trees, as per Vroman et al. (2010), are cleared and maintained at levels below 5% cover. Future climate according to scenario 11. |
| **Storm-wave** | 14 | BASELINE: Current beach slope; current climate | | Wave run-up elevations resulting from current offshore wave climate from National Centre for Environmental Prediction (NCEP) for a 1:10 year period on a south-south westerly swell, and spring high tide levels (NCEP 2013). Beach slope calculated from the distance to the 20 m depth contour obtained from the South African Navy’s bathymetric charts and available coarse topographic data (5m contour intervals). |
|  | 15 | 3° increase in beach slope; current climate | | Anthropogenic effects on coastal erosion simulated by assuming a 3° increase in beach slope, which was used as input into the Nielsen and Hanslow (1991) wave run-up model. Climate data as for scenario 14. |
|  | 16 | Current beach slope; future climate | | Beach slope as for scenario 14. Future climate simulated using a 0.5 m rise in sea-level based on literature reviews of recent publications (Rossouw and Theron 2012; Parris et al. 2012), and by applying a 6% increase to offshore extreme waves based on regional projections from metocean climate modelling (Mori et al. 2010). |

**References**

AGIS (2007) Agricultural Geo-Referenced Information System. Available: http://www.agis.agric.za. Accessed August 2013.

Hughes DA, Mantel SK, Slaughter A (2011) Developing climate change adaptation measures and decision support system for selected South African water boards. Water Research Commission Report K5/2018/2, Water Research Commission, Pretoria.

Hughes DA (2004) Problems of estimating hydrological characteristics for small catchments based on information from the South African national surface water resource database. Water SA 30: 393-398.

Le Maitre D, van Wilgen BW, Chapman RA, McKelly DH (1996) Invasive plants in the Western Cape, South Africa: modelling the consequences of a lack of management. J Appl Ecol 33: 161-172.

Lynch SD (2004) Development of a raster database of annual, monthly and daily rainfall for southern Africa. Report No. 1156/1/04, Water Research Commission, Pretoria.

Middleton BJ, Bailey AK (2009) Water Resources of South Africa 2005 Study. Water Research Commission Report Number TT 380/08, Water Research Commission, Pretoria.

Mori N, Yasuda T, Mase H, Tom T, Oku Y (2010) Projection of extreme wave climate change under global warming. Hydrological Research Letters 4: 15-19.

NCEP (2013) Offshore data on wave height, period and direction. Available: http://www.nco.ncep.noaa.gov/pmb/products/wave/#WW3ENS. Accessed August 2013.

Nielsen P, Hanslow DJ (1991) Wave run-up distributions on natural beaches. J Coastal Res 7: 1139-1152.

Parris A, Bromirski P, Burkett V, Cayan D, Culver M, et al. (2012). Global Sea Level Rise Scenarios for the US National Climate Assessment. NOAA Tech Memo OAR CPO-1. 37 p.

Rossouw M, Theron AK (2012) Investigation of potential climate change impacts on ports and maritime operations around the southern African coast. In: Asariotis R, Benamara H, editors. Maritime Transport and the Climate Change Challenge. Routledge: United Nations Conference on Trade and Development (UNCTAD), Earthscan. 360 p.

Scott DF (1993) The hydrological effects of fire in South African mountain catchments. J Hydrol 150: 409-432.

Scott D F, Prinsloo FW, Moses G, Mehlomakulu M, Simmers ADA (2000). A Re-analysis of the South African Catchment Afforestation Experimental Data. Water Research Commission Report Number 810/1/00, Water Research Commission, Pretoria.

Shakesby RA, Doerr SH (2006) Wildfire as a hydrological and geomorphological agent. Earth-Sci Rev 74: 269-307.

van den Berg EC, Plarre C, van den Berg HM, Thompson MW (2008) The South African National Land Cover 2000. Agricultural Research Council (ARC) and Council for Scientific and Industrial Research (CSIR), Report No. GW/A/2008/86, Pretoria.

van Wilgen BW, Richardson DM (1985) The effect of alien shrub invasions on vegetation structure and fire behaviour in South African fynbos shrublands: a simulation study. J Appl Ecol 22: 955 966.

Vromans DC, Maree KS, Holness S, Job N, Brown AE (2010) The Garden Route Biodiversity Sector Plan for the George, Knysna and Bitou Municipalities: Supporting land-use planning and decision-making in Critical Biodiversity Areas and Ecological Support Areas for sustainable development. Garden Route Initiative. South African National Parks, Knysna.
